# Exploring the avian gut microbiota: current trends and future directions

**DOI:** 10.3389/fmicb.2015.00673

**Published:** 2015-07-03

**Authors:** David W. Waite, Michael W. Taylor

**Affiliations:** Centre for Microbial Innovation, School of Biological Sciences, University of AucklandAuckland, New Zealand

**Keywords:** bird, avian, microbiota, bacteria

## Abstract

Birds represent a diverse and evolutionarily successful lineage, occupying a wide range of niches throughout the world. Like all vertebrates, avians harbor diverse communities of microorganisms within their guts, which collectively fulfill crucial roles in providing the host with nutrition and protection from pathogens. Across the field of avian microbiology knowledge is extremely uneven, with several species accounting for an overwhelming majority of all microbiological investigations. These include agriculturally important birds, such as chickens and turkeys, as well as birds of evolutionary or conservation interest. In our previous study we attempted the first meta-analysis of the avian gut microbiota, using 16S rRNA gene sequences obtained from a range of publicly available data sets. We have now extended our analysis to explore the microbiology of several key species in detail, to consider the avian microbiota within the context of what is known about other vertebrates, and to identify key areas of interest in avian microbiology for future study.

## Introduction

Birds represent a diverse and evolutionarily successful lineage, with over 10,000 extant species (Gill and Donsker, 2015) ranging from the diminutive bee hummingbird (weighing in at a mere 2 g) to the towering ostrich (>100 kg and >2 m tall). Such extreme morphological diversity is mirrored by the wide range of lifestyles adopted by avians, with the capacity of most species to fly facilitating the colonization of niches in ecosystems throughout the world. Bird diets vary widely, from fruit, seeds and foliage through to carrion and the capture of live animals. In this review article, we consider some of the challenges imposed on birds by their diet and lifestyle, and explore the potential relevance of gut microorganisms in assisting them to deal effectively with such constraints.

The gastrointestinal (GI) tracts of birds—like those of other vertebrate hosts—harbor a community of microbes, with densities as high as 10^11^ CFU/g in the hindgut (Barnes, 1972). While there is extensive evidence that microbial colonization of the GI tract brings benefits to host birds (Jin et al., 1998; Torok et al., 2008, 2011; Angelakis and Raoult, 2010; Zhang et al., 2011; Cao et al., 2012; Stanley et al., 2012), there are also pathways through which the normal colonization of microbes can be of detriment (Ford and Coates, 1971; Potti et al., 2002; Cao et al., 2012; Singh et al., 2013). Our knowledge of the avian microbiota has arguably lagged behind that of many other vertebrates, most notably humans (Turnbaugh et al., 2007) and mice (Benson et al., 2010; McKnite et al., 2011; Campbell et al., 2012; Hildebrand et al., 2013) but also other mammals, insects, and even fish (Sullam et al., 2012; Engel and Moran, 2013). In recent years, however, the data generated in avian microbiology have markedly increased (Supplemental Table S1, Supplemental Figure S1). It is now evident that the gut microbiota influences the health and physiology of vertebrate hosts, with recognized roles for the vertebrate microbiota in nutrition, gut development and regulation of host physiology.

Technological advances, most notably the advent of next-generation sequencing platforms, such as 454 GS FLX pyrosequencing and the Illumina HiSeq/MiSeq, have reduced the costs of sequencing by orders of magnitude, enabling unprecedented insights into both the diversity and function of microbes within the vertebrate GI tract. Large-scale sequencing of 16S rRNA genes from GI bacteria and archaea has been particularly profitable, with recent efforts to infer functional characteristics from 16S rRNA data also showing real promise (Zaneveld et al., 2010; Muegge et al., 2011; Langille et al., 2013). Our recent article (Waite and Taylor, 2014) represented the first attempt to unify the current data pertaining to avian microbiology into a single meta-analysis and to reveal the influence of environment and lifestyle on the avian microbiota.

**Figure 1:**
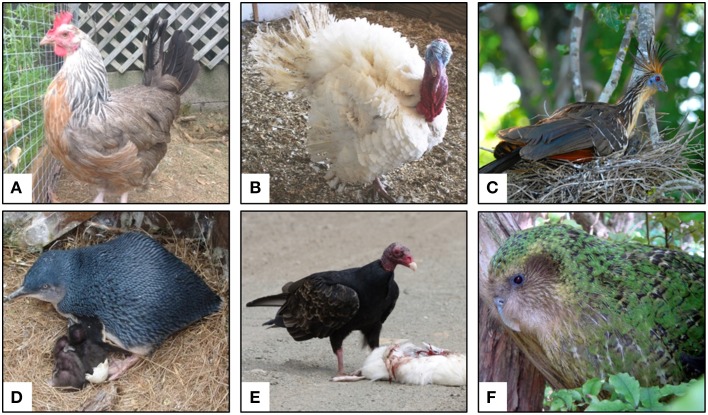
**A selection of avians that have been studied in a microbiology context**. Chicken (*Gallus gallus domesticus*), **(A)**; turkey (*Meleagris gallopavo*), **(B)**; hoatzin (*Opisthocomus hoazin*), **(C)**; little penguin (*Eudyptula minor*), **(D)**; turkey vulture (*Cathartes aura*), **(E)**; and the kakapo (*Strigops habroptilus*), **(F)**. All images are used with the authors' permission, as detailed in the Acknowledgments.

The revolution in 16S rRNA gene sequencing has seen earlier studies of microbial physiology within birds complemented by investigations into the diversity and phylogeny of avian gut microbes. Research foci have recently included the variation in microbial community structure along the GI tract, the effect of diet and age, and for some hosts the influence of factors such as captivity, antibiotic treatment, or pathogen colonization (Table 1) (Waite and Taylor, 2014). While commercially important bird species such as broiler chickens and turkeys have long received attention from microbiologists, other host avians such as the folivorous hoatzin, carnivorous penguins, scavenging vultures, and critically endangered kakapo have also come under recent scrutiny (Figure 1). Here we re-examine existing knowledge of the avian microbiota, particularly within the context of the wider vertebrate microbiota, and identify the key outstanding questions in avian microbiology.

**Table 1:** **Summary of studies investigating the impact of biological and non-biological factors on the avian microbiota**.

**Factor**	**Host**	**Study**
Variation along the GI tract	Chicken	Bjerrum et al., 2006
		Gong et al., 2007
	Turkey	Torok et al., 2008
	Hoatzin	Godoy-Vitorino et al., 2012
	Kakapo	Waite et al., 2012
Host age	Chicken	Van Der Wielen et al., 2002
		Lu et al., 2003
		Scupham, 2007
	Hoatzin	Godoy-Vitorino et al., 2010
	Kittiwake	Van Dongen et al., 2013
Dietary and probiotic manipulation	Chicken	Jin et al., 1998
		Rubio et al., 1998
		Blanco et al., 2006
		Janczyk et al., 2009
		Hammons et al., 2010
		Torok et al., 2011
		Stanley et al., 2012
		Dewar et al., 2014b
Captivity and antibiotic treatment	Capercaillie	Wienemann et al., 2011
	Kakapo	Waite et al., 2014
	Turkey	Scupham et al., 2008
	Parrots	Xenoulis et al., 2010
	Penguin	Singh et al., 2013

Note that many of these studies investigate multiple facets of the microbiota so may overlap with other categories.

## Different lifestyles, different challenges

The plethora of ecological strategies that have been adopted by avians means that, collectively, birds have to cope with a wide range of gastronomic challenges. This is reflected in the large variation in organ morphology among different bird species, although despite these often substantial differences the only avian lineage showing major differences from the “standard” gut layout is that of the psittacines (parrots), which lack ceca (Stevens and Hume, 1998). The ceca are a pair of finger-like appendages protruding from the junction of the small and large intestine which facilitate nitrogen cycling, carbohydrate fermentation and aid water retention (McNab, 1973; Mead, 1989; Józefiak et al., 2004). The nutritional characteristics of different bird diets vary greatly, while toxins of both plant and carrion origin may also be encountered by certain species. While inter-individual variation exists within the avian **microbiome**, this effect is overshadowed by factors such as diet or host age. As shown for other vertebrates (Ley et al., 2008), one would expect the GI **microbiota** of avians to reflect their particular lifestyle. Here, we consider some of the better-studied avian hosts and their diets and seek to illustrate the enabling role played by gut microbes within these diverse species.

KEY CONCEPT 1 **Microbiota vs. microbiome**

Although sometimes used synonymously, these terms are generally meant to describe subtly different things. Microbiota refers to the community of microorganisms within a given environment (e.g., the avian gut), while microbiome describes the collective genomes of all these microbial species.

The hoatzin (*Opisthocomus hoazin*; Figure 1) of South America is something of a biological anomaly. Unlike most birds, it feeds mostly on leaves and—uniquely among avians—carries out foregut fermentation in an enlarged crop (Grajal et al., 1989), making its digestive strategy more like that of a ruminant than a bird. Indeed, the hoatzin foregut microbiota—dominated by members of the *Bacteroidetes*, *Firmicutes*, and *Proteobacteria*—is more similar to that of a cow's rumen than it is to its' own hindgut microbiota, demonstrating the importance of organ function in determining microbial community composition (Godoy-Vitorino et al., 2012). The hoatzin crop and cow rumen thus represent an excellent example of evolutionary convergence: a diet dominated by plant fibers requires a fermentation chamber of sufficient volume to retain this refractory material until its microbial breakdown into host-assimilable products such as short-chain fatty acids. Mechanical considerations predict that such an enlarged crop would preclude flying, and yet the hoatzin is capable of flight. This may be due to the highly selective diet that flying affords, allowing the hoatzin to specifically target higher quality foods with consequent rapid fermentation and optimal digestive efficiency (Grajal et al., 1989). Crop bacteria may also perform another important role for the hoatzin, by degrading toxic polyphenolic compounds which are present in many of the consumed plant species (Domínguez-Bello et al., 1994; Garcia-Amado et al., 2003, 2007). The extent to which the hoatzin relies on behavioral (avoidance) strategies, or its own crop microbiota, to enable its folivorous diet remains uncertain (Jones et al., 2000).

Another ecological niche which exposes the bird to toxic components is that exploited by vultures. Vultures, such as the turkey vulture (*Cathartes aura*; Figure 1) and black vulture (*Coragyps atratus*), consume carrion, feeding upon decaying animal carcasses that are in some cases days old. These habits expose vultures to a range of potential pathogens (Marin et al., 2014; Sulzner et al., 2014) as well as bacterial toxins, such as botulinum, produced during tissue breakdown. This is enough to deter most potential consumers, yet vultures are seemingly inured to these compounds. The faces of turkey and black vultures, which are often inserted inside decaying body cavities of vertebrate prey species, contain a highly diverse, yet substantially overlapping microbiota consisting primarily of *Actinobacteria*, *Firmicutes* (*Bacilli* and *Clostridia*), as well as *Beta*- and *Gammaproteobacteria*. The similarity in microbiota between the two vulture species likely reflects the common diets of these co-occurring scavengers (Roggenbuck et al., 2014). Corresponding hindgut samples yielded significantly lower bacterial diversities (though with some overlapping phylotypes), indicating that most diet-derived bacteria do not survive the passage from the mouth to the gut. The hindgut microbiota of both vulture species is dominated by members of only two bacterial classes, *Clostridia* and *Fusobacteria* (Roggenbuck et al., 2014). It is surmised that these two taxa outcompete other bacteria in the vulture hindgut, while the bird can tolerate bacterial toxins in order to exploit their degradation of carrion tissues.

The kakapo (*Strigops habroptilus*; Figure 1) of New Zealand is the world's heaviest parrot and only flightless parrot. It is also the only parrot that performs a mating ritual known as lek breeding, through which males compete for mates through a characteristic booming call. It is, unfortunately, also one of the world's rarest species, with only 126 individuals confined to three predator-free islands off New Zealand's coast. Like the aforementioned hoatzin, the kakapo is herbivorous, and it has long been speculated that it also performs foregut fermentation (Morton, 1978). However, the two bird species are markedly different when it comes to feeding strategy. The kakapo typically pulls foliage through its beak, sucking out nutrients while leaving the more recalcitrant, fibrous material behind in characteristic “chews” (Oliver, 1955; Horrocks et al., 2008). The requirement for an extensive microbial community to ferment plant material is therefore dramatically reduced, as reflected in recent molecular studies which revealed the kakapo microbiome to typically be dominated (up to ~95%) by only two bacterial phylotypes belonging to the genera *Escherichia* and *Streptococcus* (Waite et al., 2013, 2014).

The study of microbial diversity and function in avians is a burgeoning field, with recent focus not only on the birds mentioned above, but also others including penguins (Figure 1) and commercially important species such as chickens and turkeys (Figure 1). Unsurprisingly, commercially raised fowl have long been the subject of microbiological investigation, but their microbiology has been reviewed elsewhere (Yeoman et al., 2012; Wei et al., 2013; Oakley et al., 2014; Cox and Dalloul, 2015; Deusch et al., 2015) and is not specifically covered here.

## What do we know about the avian gut microbiota?

**Figure 2:**
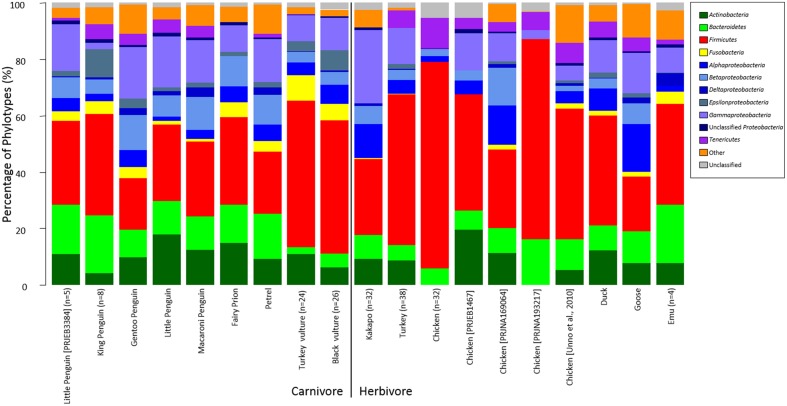
**Relative proportions of bacterial phylotypes detected in the avian gut microbiota**. Data were processed as per Waite and Taylor (2014). Figure displays the number of genus-level phylotypes detected in each sample, not the relative abundance of these phylotypes. Where studies analyzed multiple individuals, results were averaged across the study with the number of individuals in brackets. Data sources are as detailed in Supplemental Table S1.

**Figure 3:**
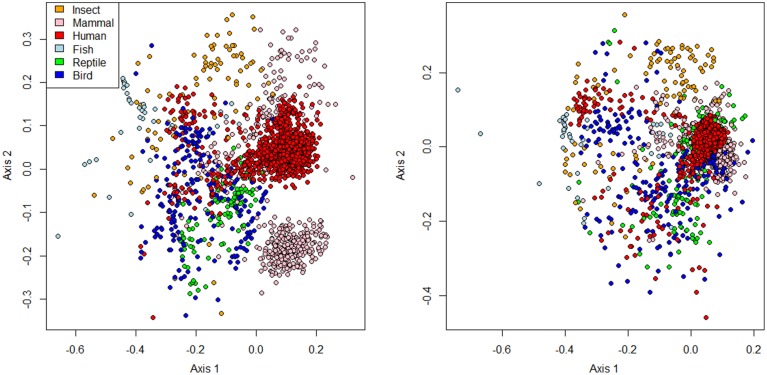
**Non-metric multidimensional scaling plots of the avian gut microbiota compared to that of other animal lineages**. Data from next-generation amplicon sequencing sources were used. Figure displays NMDS based on unweighted unifrac distance (left, stress = 0.18, *R*^2^ = 0.87) and Jaccard dissimilarity index of genus-level phylotypes (right, stress = 0.19, *R*^2^ = 0.91). Unweighted UniFrac distances were inferred through closed-reference OTU picking using QIIME 1.80, and phylotypes were generated using the assign_taxonomy.py script with the Greengenes database (May 2013 release) (Caporaso et al., 2010). Three dimensional representations of the data are provided in Supplemental Figures S2, S3. All data sources are provided in Supplemental Table S2.

Much like for other vertebrate hosts, the GI microbiota of avians is dominated by members of the *Firmicutes*, with *Actinobacteria*, *Bacteroidetes*, and *Proteobacteria* also commonly observed (Figure 2). This broad generalization holds across both herbivores and carnivores, although the relative proportions of these groups can vary substantially. Despite these superficial similarities, the microbiota of avians is somewhat distinct from that of other branches of the tree of life. Hird et al. (2014) compared the microbiota of numerous avians to a larger sequence data set (Ley et al., 2008) and showed that the gut microbiota of avians clustered apart from that of mammals and insects. Performing this analysis with our own aggregation of data also demonstrated this finding (Figure 3, Supplemental Figures S2, S3), with avian samples showing clustering apart from samples obtained from humans and other mammals, insects and fish (ANOSIM, 0.49 < R < 0.72) but surprisingly weak structuring apart from samples obtained from reptiles (ANOSIM *R* = 0.12) (*p* < 0.001 for all comparisons). Among avians, the richness of the microbiota can vary considerably, ranging from dominance by only a handful of phylotypes, as in the case of the kakapo, to a highly diverse community comprising 40 bacterial phyla, as observed for the hoatzin (Godoy-Vitorino et al., 2010) (the hoatzin data were not included in Figure 2 due to differences in the methodology by which they were obtained (PhyloChip microarray vs. next-generation sequencing)). For a more detailed description of the microbiota data, including diversity comparisons (with an average of 5.5 bacterial phyla per avian sample), the reader is referred to our recent meta-analysis paper (Waite and Taylor, 2014). The functional role of microbes in the hindgut of birds has historically been an area of interest (Bolton, 1965; Pritchard, 1972; Józefiak et al., 2004), with microbial production of lactate, acetate and other short-chain fatty acids strongly implicated in the health of birds and other vertebrates (Van Der Wielen et al., 2000; Hosseini et al., 2011; Fukuda et al., 2012). More recently, the focus has moved to areas of greater interest in agriculture, predominantly the roles of microbial strains or communities in preventing pathogen colonization and for boosting weight gain (Watkins and Miller, 1983; Jin et al., 1998; Cutler et al., 2005; Torok et al., 2008; Zhang et al., 2011, 2014; Cao et al., 2012; Chen et al., 2013).

Although **host genetics** clearly influence the human gut microbiota (Goodrich et al., 2014) and evidence exists for the importance of host-specific factors as determinants of the avian gut microbiota (Zhu et al., 2002; Banks et al., 2009; Benskin et al., 2010; Grond et al., 2014), the scale at which this effect is observed is small compared to that of other factors such as diet (Lozupone et al., 2013; Carmody et al., 2015). A recent analysis by Hird et al. (2014) addressed the influence of environmental and host factors on shaping the avian microbiota by comparing fecal microbiota profiles of the cowbird (an avian brood parasite) to those of birds known to host cowbird young. The data obtained showed clearly that location, age and diet all play greater roles in shaping the gut microbiota of cowbirds than the taxonomic identity of the birds studied. A similar result was reported for members of the Paridae in an earlier study (Lucas and Heeb, 2005) and has also been shown for chickens raised in captivity (Stanley et al., 2013). While gut microbes convey benefits to their hosts in a variety of ways (Pritchard, 1972; Domínguez-Bello et al., 1993; Preest et al., 2003; Shawkey et al., 2007; Burtt et al., 2011), the findings of Hird and colleagues suggest that the microbiota is more a product of the environment than a trait of the bird itself. Our own aggregation of data support this finding, with occurrence of bacterial phyla (*Actinobacteria*, *Fusobacteria*) and classes (*Alpha*-, *Beta*-, *Delta*-, and *Epsilonproteobacteria*) varying between different studies of the same host organism (Figure 2, chicken). Our meta-analysis of **amplicon sequence** data also revealed study origin to have greater explanatory power than biological variables, such as diet, when investigating the avian microbiota (Waite and Taylor, 2014). The interplay between environmental factors and host genetics in shaping the microbiome has been reported for other vertebrates (Benson et al., 2010; Campbell et al., 2012; Stanley et al., 2013), although care must be taken when dissecting genetic effects and the role of maternal inoculation of the microbiota (Spor et al., 2011).

KEY CONCEPT 2 **Host genetics**

The influence of host genetics on the gut microbiota is an intriguing, and complicated, facet of microbiology. Host genetics have a measurable, albeit subtle, influence on the gut microbiota at both inter-species and within-population resolutions, likely resulting from physiological differences between the guts and immunological profiles of individuals. However, these effects are often overshadowed by other environmental factors, with cohabitation and local diet patterns providing confounding influences.

KEY CONCEPT 3 **Amplicon sequencing**

A generic term to describe the PCR amplification, and subsequent sequencing by one of the “next-generation” sequencing technologies, of genes from extracted microbial DNA. Amplicon sequencing most commonly utilizes 16S ribosomal RNA (rRNA) genes, as these are conserved in all bacteria and archaea (eukaryotes contain the homologous 18S rRNA gene). The analysis of rRNA genes via sequencing has been a cornerstone of microbial ecology for more than two decades, while functional genes (e.g., encoding for specific enzymes) can also be targeted with the amplicon sequencing approach.

An interesting aspect of avian microbiology that has only recently come to light is the potential role of *Fusobacteria* in the guts of carnivorous birds. While members of the *Fusobacteria* are often studied in the context of pathogenicity, recent analyses of the vulture microbiota have revealed abundant populations of *Fusobacteria* that appear to be beneficial, or at the very least harmless, to the host bird (Roggenbuck et al., 2014). While *Firmicutes*, *Bacteroidetes* and *Proteobacteria* are the most consistently observed bacterial phyla across the animal gut microbiota, a rich community of *Fusobacteria* has frequently been reported in the guts of carnivorous and omnivorous avians. These include close relatives of the human pathogens *Fusobacterium nucleatum* and *F. necrophorum*, while the avian pathogen *Streptobacillus moniliformis* (also a member of the phylum *Fusobacteria*) was detected in some penguin samples. Up to one third of the vulture gut microbiota, and over half of the penguin microbiota, can consist of *Fusobacteria* (Dewar et al., 2013, 2014a; Roggenbuck et al., 2014; Vela et al., 2014), with *F. mortiferum* predominating among *Fusobacteria* within the vulture microbiota. *Fusobacteria* are also observed at a lower abundance in other carnivorous seabirds and the omnivorous bustard (Dewar et al., 2014b; Shabbir et al., 2014), and have been reported in the guts of some non-mammalian carnivores (Keenan et al., 2013; Nelson et al., 2013), although a consistent pattern has not been observed in mammals (Ley et al., 2008; Swanson et al., 2011; Nelson et al., 2013). While the consistent appearance of *Fusobacteria* in the avian microbiome is an interesting avenue for further study, the potential role of this phylum in avian nutrition requires further analysis, as does the occurrence of *Fusobacteria* in captive avians (Figure 2).

## A look to the future

**Figure 4:**
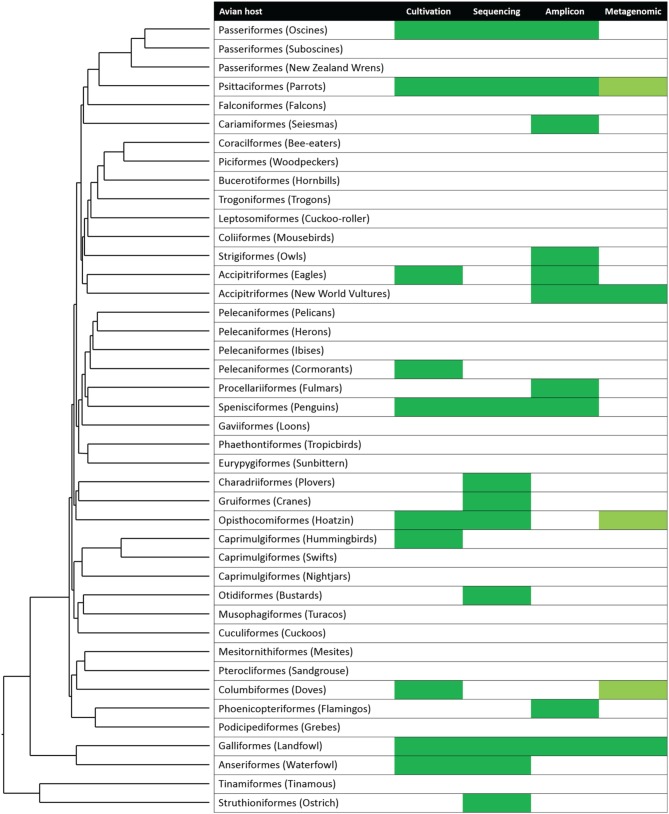
**Coverage of microbiological representation across avian lineages**. Two studies have been performed on avian orders with no representation in the displayed phylogeny (emu, Bennett et al., 2013; cormorant, Tausova et al., 2012). The microbiota of several species has been studied using molecular techniques other than sequence analysis (Lucas and Heeb, 2005; Ruiz-Rodríguez et al., 2009). Pale green indicates data that are available on online repositories, but currently unpublished. Figure modified from Jarvis et al. (2014); reprinted with permission from AAAS and the author.

The gut microbiology of humans and other vertebrates has exploded as a discipline over the past decade, driven by technological advances and an increased appreciation of the vital roles played by the gut microbiome in animal health. Although study of the avian microbiota has arguably lagged behind that of some other host organisms, this gap is closing rapidly. As discussed within these pages, certain key taxa—such as the chicken, turkey, hoatzin, and kakapo—have received considerable research attention from microbiologists, and it is to be hoped that our current knowledge of the avian microbiota will soon be extended to encompass many of the “missing” branches of the bird tree of life (Figure 4). Expanding the knowledge on any avian lineage will be of value in resolving high-level patterns of the influence of diet and lifestyle on the gut microbiota. A lineage that we feel would be of great interest is the hummingbird, which possesses the fastest metabolism among homeothermic animals (Suarez, 1992) and relies on a diet primarily of nectar supplemented with arthropods (Brice, 1992; Yanega and Rhubega, 2004; Powers et al., 2010). As an adaptation to the extreme levels of sugar ingested, hummingbirds possess extremely potent sucrase (Martínez Del Rio, 1990) and are able to survive in a constant state of hyperglycemia (Beuchat and Chong, 1998). Although some study of bacterial activity in hummingbirds has been performed (Preest et al., 2003), deeper analysis of this community would greatly aid our understanding of not only gut bacteria in general but specifically life in an osmotically challenging environment.

Despite containing the same core phyla as the mammalian gut microbiota, the “typical” microbiota of avians is clearly distinct from that of mammals and insects (Hird et al., 2014) and analysis of full-length 16S rRNA genes often yields sequences that diverge heavily from previously obtained bacterial sequences (Zhu et al., 2002; Bjerrum et al., 2006; Godoy-Vitorino et al., 2008, 2010). The pace at which 16S rRNA gene information has grown has left many other aspects of microbial genomics and ecology behind. The content of the publicly available 16S rRNA gene sequences in the Greengenes database outnumbers prokaryotic type strains by over 40:1, and prokaryotic genomes by about 20:1 (Kyrpides et al., 2014). While some inferences about the functional roles of microbes associated with the avian GI tract can be made using predictive software such as PICRUSt (Langille et al., 2013), much of the actual function of these communities remains unclear, especially when considering the significant evolutionary specialization which gut-associated bacteria often undergo (Comstock and Coyne, 2003; Xu et al., 2003; Meinl et al., 2009; Frese et al., 2011; Foley et al., 2013). The suite of meta-“omics” techniques offers partial solutions to this issue obviating, or in some cases aiding, the need for cultivation and potentially providing new insights into the community-level function and ecological interactions in the avian gut. While not related to the study of avians, metagenomics has previously been applied to tailor cultivation conditions in order to isolate previous uncultivated bacteria (Tyson et al., 2004, 2005). One can easily envisage the complementary application of metagenomics, metatranscriptomics and metaproteomics in order to elucidate the functional potential, gene expression and protein production, respectively, of the microbial community associated with an avian host. Such integrated approaches have been applied to gut environments to some extent (Pérez-Cobas et al., 2013; Kato et al., 2014) and, when used in combination with recently acquired bird genome data (Jarvis et al., 2014), would offer unprecedented insights into avian-microbe interactions.

Our own studies of the kakapo microbiome (Waite et al., 2013, 2014) have identified an area of potential wider research and applied interest, namely the use of microbial ecology approaches to aid in avian conservation. To use the kakapo as a case in point, characterization of the indigenous microbiota should facilitate the detection of microbial dysbiosis and, potentially, the identification of invading pathogenic species, while longitudinal studies have allowed the effects of human intervention and intrinsic host characteristics on the gut microbiota to be teased apart (Waite et al., 2014). Such an approach should be equally beneficial for the conservation of any intensively managed, endangered bird.

Studying the impact of environmental factors on the avian gut microbiota highlights another area in which avian microbiology is lacking, namely the existence of a global, unified initiative using a common methodology to resolve large-scale patterns in microbial distribution and function. The recently completed Human Microbiome Project (Human Microbiome Project Consortium, 2012; Human Microbiome Project, 2014) represented a landmark in microbiology, bringing together research from a number of institutions and research groups to tackle a series of overarching questions. Following in the footsteps of the HMP, a range of projects have been enacted to address areas of ecological and medical microbiology in a standardized manner. The Earth Microbiome Project (Gilbert et al., 2014), Hospital Microbiome Project (http://hospitalmicrobiome.com/) and 1000 Springs project (http://www.1000springs.org.nz/) all aim to study large sources of data using a common laboratory and bioinformatics approach in order to reduce methodological artifacts, which are known to contribute large sources of bias to studies. Other studies have begun to combine genome-wide and microbiome-wide association studies to comprehensively identify the genetic pathways through which vertebrate hosts influence the microbiota (Benson, 2014).

In summary, while the avian microbiota is an area of microbiology of great economic importance and scientific interest, the field has arguably not advanced as quickly as some other areas of microbiology. While trends seen in avian microbiology do appear to reflect those seen in other vertebrates there are key areas of the avian microbiota that distinguish it from that of mammals and reptiles. The last decade has seen a massive leap forward in the technology available to microbiologists and while the avian microbiota has lagged behind other vertebrates, interest appears to be growing in this area of microbiology and many novel avenues of study exist within avian microbiology.

### Conflict of interest statement

The authors declare that the research was conducted in the absence of any commercial or financial relationships that could be construed as a potential conflict of interest.

## Supplementary material

Supplemental Figure S1 **Cumulative number of 16S rRNA gene data sets from published research investigating the avian microbiota**. Red line denotes the addition of 16S rRNA gene data obtained from next-generation sequencing platforms to the main data set. Counts incorporating these sequence read archive (SRA) data (red line) are inclusive of full-length 16S rRNA gene sequence data. Published studies were identified using the SCOPUS database, and additional SRA data through the NCBI SRA website using the search string “(bird OR avian OR aves) AND microb^*^.” All returned studies were manually examined to ensure they had submitted sequence data to a publically available repository.

Supplemental Figure S2 **Three dimensional non-metric multidimensional scaling plot of the unweighted UniFrac distances between samples**. Unweighted UniFrac distances were inferred through closed-reference OTU picking using QIIME 1.80 (Caporaso et al., 2010) against the Greengenes 16S rRNA gene database (May 2013 release). Plot was visualized using the “rgl” package in R.

Supplemental Figure S3 **Three dimensional non-metric multidimensional scaling plot of the binary Jaccard distances of genus-level phylotypes between samples**. Phylotypes were constructed following classification using the assign_taxonomy.py script in QIIME 1.80 (Caporaso et al., 2010) against the Greengenes 16S rRNA gene database (May 2013 release). Plot was visualized using the “rgl” package in R.

Supplemental Table S1 **Summary of published sequence data obtained from molecular analysis of avian samples**. Asterisk (*) denotes a study that analysed the bacterial communities associated with multiple species of birds. Cultivation studies are only included where they utilised non-selective cultivation conditions (e.g. did not select for antibiotic-resistant bacterial strains). Where publication details associated with a data set could not be obtained the NCBI Sequence Read Archive BioProject accession is provided.

Supplemental Table S2 **16S rRNA amplicon data sources for comparative microbiota analysis**. All data were quality filtered as described in Waite and Taylor (2014) prior to analysis.
