# Supplementary figures and images for: Exploring the avian gut microbiota: current trends and future directions
Source: Front Microbiol. 2015 Jul 3;6:673. doi: 10.3389/fmicb.2015.00673 (PMC4490257; doi:10.3389/fmicb.2015.00673)

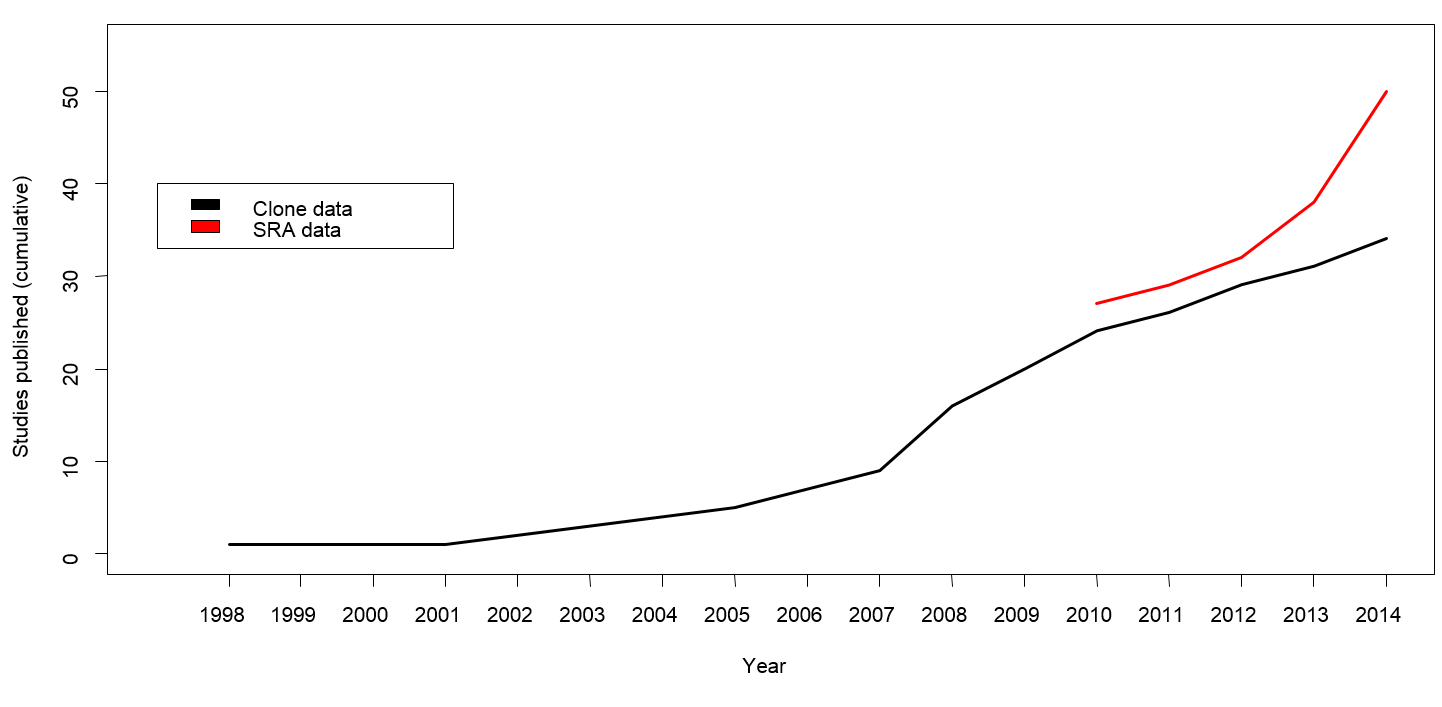

Supplement: Supplemental Figure S1 — Cumulative number of 16S rRNA gene data sets from published research investigating the avian microbiota. Red line denotes the addition of 16S rRNA gene data obtained from next-generation sequencing platforms to the main data set. Counts incorporating these sequence read archive (SRA) data (red line) are inclusive of full-length 16S rRNA gene sequence data. Published studies were identified using the SCOPUS database, and additional SRA data through the NCBI SRA website using the search string “(bird OR avian OR aves) AND microb*.” All returned studies were manually examined to ensure they had submitted sequence data to a publically available repository. [file Image1.TIF]
