# Supplementary material for: Exploring the avian gut microbiota: current trends and future directions
Source: Front Microbiol. 2015 Jul 3;6:673. doi: 10.3389/fmicb.2015.00673 (PMC4490257; doi:10.3389/fmicb.2015.00673)
Supplement: Supplemental Figure S3 — Three dimensional non-metric multidimensional scaling plot of the binary Jaccard distances of genus-level phylotypes between samples. Phylotypes were constructed following classification using the assign_taxonomy.py script in QIIME 1.80 (Caporaso et al., 2010) against the Greengenes 16S rRNA gene database (May 2013 release). Plot was visualized using the “rgl” package in R. [file Presentation2.ZIP › Figure S3.html]

Supplemental Figure S3


## Non-metric multidimensional scaling plot of the binary Jaccard distances between individual samples.

Exported from R using rgl 0.93.996 by writeWebGL.  
Drag mouse to rotate model. Use mouse wheel or middle button to zoom.

---

Your browser does not support the HTML5 canvas element.


You must enable Javascript to view this page properly.

|  |  |
| --- | --- |
|  | Insect |
|  | Mammal |
|  | Fish |
|  | Human |
|  | Reptile |
|  | Bird |
| Stress: 0.14 | |
| R2: 0.94 | |
