# Supplementary material for: Exploring the avian gut microbiota: current trends and future directions
Source: Front Microbiol. 2015 Jul 3;6:673. doi: 10.3389/fmicb.2015.00673 (PMC4490257; doi:10.3389/fmicb.2015.00673)
Supplement: Supplemental Table S1 — Summary of published sequence data obtained from molecular analysis of avian samples. Asterisk (*) denotes a study that analysed the bacterial communities associated with multiple species of birds. Cultivation studies are only included where they utilised non-selective cultivation conditions (e.g. did not select for antibiotic-resistant bacterial strains). Where publication details associated with a data set could not be obtained the NCBI Sequence Read Archive BioProject accession is provided. [file DataSheet1.DOCX]

**Supplemental Table S1.** **Summary of published sequence data obtained from molecular analysis of avian samples.**

Asterisk (*) denotes a study that analysed the bacterial communities associated with multiple species of birds. Cultivation studies are only included where they utilised non-selective cultivation conditions (e.g. did not select for antibiotic-resistant bacterial strains). Where publication details associated with a data set could not be obtained the NCBI Sequence Read Archive BioProject accession is provided.

| **Host** | **Site sampled** | **Method of analysis** | **Reference** |
| --- | --- | --- | --- |
| Adelie penguin | Faecal | Clone library | Banks et al. (2009) |
| Bobwhite | Trachea/Crop/Ceca/Cloaca | Cultivation | Su et al. (2014) |
| Bustard | Trachea | Amplicon | Shabbir et al. (2014) |
| Capercaillie | Cecum | Clone library | Wienemann et al. (2011) |
| Chicken | Cecum | Clone library | Zhu et al. (2002) |
|  | Illeum/Cecum | Clone library | Lu et al. (2003) |
|  | Cecum | Clone library | Bjerrum et al. (2006) |
|  | Trachea | Cultivation | Tsai and Huang (2006) |
|  | Crop/Cecum | Clone library | Gong et al. (2007) |
|  | Faecal | Amplicon | Unno et al. (2010) |
|  | Cecum | Amplicon | Stanley et al. (2013) |
|  | Cecum | Clone library | Torok et al. (2011) |
|  | Aggregate | Clone library and cultivation | Wei et al. (2013) |
|  | Cecum | Amplicon | PRJNA193217 |
|  | Ileum | Amplicon | PRJEB1467 |
|  | Faecal | Amplicon | PRJNA169064 |
| *Corvus* sp.* | Cloaca | Cultivation | Jansson et al. (2008) |
| Cowbird* | Faecal | Amplicon | Hird et al. (2014) |
| Crane | Faecal | Clone library | Ryu et al. (2012) |
| Duck | Crop/ceca | Cultivation | Kurzak et al. (1998) |
|  | Faecal | Amplicon | Unno et al. (2010) |
| Emu | Cecum | Amplicon | Bennett et al. (2013) |
| Goose | Faecal | Cultivation | Damere et al. (1979) |
|  | Faecal | Clone library | Lu et al. (2009) |
|  | Faecal | Amplicon | Unno et al. (2010) |
| Gull | Faecal | Clone library | Lu et al. (2008) |
| Hoatzin | Crop | Clone library | Godoy-Vitorino et al. (2008) |
|  | Crop | Clone library | Wright et al. (2009) |
|  | Crop | Clone library | Godoy-Vitorino et al. (2010) |
| Hummingbird | Intestine | Cultivation | Preest et al. (2003) |
| Kakapo | Crop/Faecal | Clone library | Waite et al. (2012) |
|  | Crop/Faecal | Amplicon | Waite et al. (2014) |
| Little Penguin | Cloaca | Amplicon | PRJEB3384 |
| Ostrich | Cecum | Clone library | Matsui et al. (2010) |
| Parrot* | Crop^C^ | Cultivation | Pacheco et al. (2004) |
|  | Cloaca | Clone library | Xenoulis et al. (2010) |
| Pigeon | Trachea^C^ | Cultivation | Tsai and Huang (2006) |
| Penguins* | Faecal | Amplicon | Dewar et al. (2013) |
|  | Faecal | Amplicon | Dewar et al. (2014a) |
| Red kite | Faecal^C^ | Cultivation | Blanco et al. (2006) |
| Shorebirds* | Cloaca | Clone library | Santos et al. (2012) |
|  | Faecal | Amplicon | Dewar et al. (2014b) |
|  | Faecal | Clone library | Grond et al. (2014) |
|  | Faecal | Clone library | Ryu et al. (2014) |
| Stork | Feathers | Clone library | Nawrot et al. (2009) |
| Swan* | Faecal | Cultivation | Damere et al. (1979) |
| Towhee | Cloaca | DGGE | Klomp et al. (2008) |
| Turkey | Cecum | Clone library | Scupham (2007) |
|  | Faecal | Clone library | Lu and Domingo (2008) |
|  | Cecum | Clone library | Scupham et al. (2008) |
|  | Aggregate | Clone library and cultivation | Wei et al. (2013) |
|  | Ileum | Amplicon | Danzeisen et al. (2013) |
| Vultures* | Faecal/skin | Amplicon | Roggenbuck et al. (2014) |
|  | Faecal | Cultivation | Vela et al. (2014) |
| Waterfowl* | Faecal | Clone library | Lu et al. (2008) |
| Zebra finch | Cloaca | DGGE | Benskin et al. (2010) |

**Supplemental Table S2. 16S rRNA amplicon data sources for comparative microbiota analysis.**

All data were quality filtered as described in Waite and Taylor (2014) prior to analysis.

| **Host organism** | **Data source** | **Published source** |
| --- | --- | --- |
| Mouse | MG-RAST (Project 657) |  |
| Snake | MG-RAST (Project 77) |  |
| Soil | MG-RAST (Project 80) |  |
| Mouse | MG-RAST (Project 83) |  |
| Mouse | MG-RAST (Project 118) |  |
| Cow | MG-RAST (Project 504) |  |
| Human | MG-RAST (Project 401) | Yatsunenko et al. (2012) |
| Mouse | MG-RAST (Project 1346) |  |
| Zebrafish | MG-RAST (Project 430) |  |
| Cow | MG-RAST (Project 1876) |  |
| Cow | MG-RAST (Project 3570) |  |
| Termite | MG-RAST (Project 5942) |  |
| Human | MG-RAST (Project 7058) |  |
| Rabbit | MG-RAST (Project 8102) |  |
| Pig | MG-RAST (Project 10834) |  |
| Misc. mammals | MG-RAST (Project 114) | Muegge et al. (2011) |
| Human | MG-RAST (Project 113) | Muegge et al. (2011) |
| Biting midge (*Culicoides sonorensis*) | MG-RAST (Project 10535) |  |
| Misc. insects from New Zealand | NCBI SRA (BioProject PRJNA225474) | Waite et al. (2015). Includes unpublished data obtained from cockroaches and termites using the methods described in manuscript. |
| Termite, cockroach, 3 outgroups | NCBI SRA (BioProject PRJNA217467) | Dietrich et al. (2014) |
| Carion beetles | Dryad (doi:10.5061/dryad.2 m854) | Kaltenpoth and Steiger (2014) |
| Termite | NCBI SRA (BioProject PRJEB1238) | Boucias et al. (2013) |
| Misc. avian sources | FigShare (http://figshare.com/articles/Hird_Cowbird_fileset/957582) | Hird et al. (2014) |
| Alligator | NCBI SRA (SRA023831 and SRA062824) | Keenan et al. (2013) |
| Seal | Dryad (doi.org/10.5061/dryad.42f2q/1) | Data published in Nelson et al. (2012). Accession information provided in Nelson et al. (2013) |
